# Effect of Dietary Antioxidants in Chronic Disease Prevention

**DOI:** 10.3390/antiox14101200

**Published:** 2025-10-02

**Authors:** Baojun Xu

**Affiliations:** Food Science and Technology Program, Department of Life Sciences, Beijing Normal-Hong Kong Baptist University, Zhuhai 519087, China; baojunxu@uic.edu.cn

Chronic diseases are a major global public health challenge, with increasing incidence and mortality rates [1]. Oxidative stress, characterized by an imbalance between oxidants and antioxidants, leading to cellular damage, is a key mechanism in their development [2]. Low levels of reactive oxygen species are necessary for many processes such as intracellular signal transduction, metabolism, immune and hypoxic responses, and transcriptional regulation. However, excessive reactive oxygen species (ROS) may be pathological and contribute to the development and progression of chronic diseases [3]. Dietary antioxidants, found in fruits, vegetables, nuts, and whole grains, can neutralize free radicals and inhibit oxidative stress [4], thus playing a crucial role in chronic disease prevention [5,6]. This Special Issue delves into the latest research findings of dietary antioxidants in chronic disease prevention, including eight research articles and two reviews, which provides insights for future research and dietary guidelines.

Huang et al. studied a multicomponent dietary supplement’s impact on tear secretion and ocular surface inflammation in dry eye syndrome (DES) patients (Contribution 1). The supplement (45 mg/day eicosapentaenoic acid, 30 mg/day docosahexaenoic acid, 30 mg/day lutein, and 1.8 mg/day zeaxanthin) significantly increased tear secretion and decreased the ocular surface disease index score after 12 weeks. Inflammatory markers IL-6 and IL-8 also decreased.

Yang et al. found that Lycii Radicis Cortex (LRC) can inhibit anaerobic bacterial proliferation and inflammatory cell infiltration in rat gingival tissues, showing anti-inflammatory effects (Contribution 2). Additionally, LRC reduces malondialdehyde levels and inducible nitric oxide synthase activity, demonstrating antioxidant properties. In addition, it can effectively prevent connective tissue degradation. LRC also decreased the receptor activator of NF-κB ligand/osteoprotegerin ratio and the number and area of osteoclasts on the alveolar bone surface, thereby inhibiting alveolar bone loss.

In a cohort study, Bonfiglio et al. found that an intake of merely 165 mg of flavonoids per day exerts a protective effect against MASLD, reducing the risk of this condition (Contribution 3).

Kim et al. demonstrated through in vitro experiments using LPS-stimulated BV2 microglial cells that *Quercus acuta* Thunb. (QA) effectively mitigates microglia-mediated neuroinflammatory responses by inhibiting NF-κB and MAPK signaling pathways and activating the Nrf2/HO-1 pathway (Contribution 4).

Sandoval et al. explored in C57BL/6 mice if oral β-carotene mitigates P4502E1 (CYP2E1) expression in ethanol-exposed subjects (Contribution 5). Their findings imply β-carotene might amplify liver damage from both low and high alcohol doses. Thus, factors like alcohol volume, exposure duration, damage regulation, and signaling pathways involved in the consumption of both alcohol and antioxidants must all be weighed.

Park et al. found that Menthae Herba (MH) reduces ROS and NF-κB-mediated inflammatory signaling pathways while upregulating CREB/Nrf2/HO-1-related antioxidant signaling in microglial cells (Contribution 6).

Zhang et al. investigated the synergistic anti-inflammatory effects of proanthocyanidin (LGSP) and camellia oil (CO) in vitro (Contribution 7). The results indicated that the combined treatment of LGSP (20 μg/mL) and CO (1 mg/mL) synergistically suppressed the production of NO, TNF-, IL-6, and ROS. The synergistic effect was attributed to their suppression of the activation of nuclear factor-κB (NF-κB) and mitogen-activated protein kinase (MAPK) signaling pathways.

Yu et al. investigated the protective effect of *Mallotus oblongifolius* polyphenols (MOP) on ethanol-induced gastric mucosal injury in rats. The results showed that MOP could increase the expression of antioxidant enzymes and decrease the expression oxidative enzymes to prevent ethanol-induced acute gastric mucosal injury. These effects may be related to the inhibition of p38/ERK/JNK phosphorylation and the activation of the Nrf2 signaling pathway by MOP (Contribution 8).

The review of You et al. recorded oxidative stress and dietary antioxidants in head and neck cancer (HNSCC) (Contribution 9). The review summarized the role of oxidative stress in carcinogenesis, particularly focusing on the three major risk factors for HNSCC, including smoking, alcohol consumption, and high-risk human papillomavirus (HR-HPV) infection. Additionally, this review points out that how nine dietary antioxidants, such as vitamin C, vitamin E, carotenoids, epigallocatechin-3-gallate (EGCG), and curcumin, mitigate ROS, influence cancer-related signaling pathways, and modulate the tumor microenvironment in the development and progression of HNSCC.

Review conducted by Zhang et al., summarizes how genistein exerts therapeutic effects by inhibiting oxidative stress. Additionally, the review focuses on the mechanisms by which genistein may combat five common diseases induced by oxidative stress, including Parkinson’s disease, Alzheimer’s disease, diabetes, cardiovascular disease, and cancer. Moreover, it evaluates strategies for enhancing the water solubility and bioavailability of genistein (Contribution 10).

This Special Issue comprises a total of 10 articles, encompassing two clinical cohort studies, three in vivo experimental studies, three in vitro experimental studies, and two reviews. These studies collectively demonstrate the significant potential of dietary antioxidants in mitigating chronic diseases through their anti-inflammatory and antioxidant properties, which highlight the importance of targeted nutritional interventions in chronic disease prevention and management. Future research should focus on elucidating the deeper mechanisms and conducting larger-scale clinical trials to further validate these promising results and optimize dietary recommendations.

## Data Availability

There are no new data for this editorial.

## References

[1] Greenberg H., Pi-Sunyer F.X. (2019). Preventing preventable chronic disease: An essential goal. Prog. Cardiovasc. Dis..

[2] Sies H. (2015). Oxidative stress: A concept in redox biology and medicine. Redox Biol..

[3] Kishi S., Nagasu H., Kidokoro K., Kashihara N. (2024). Oxidative stress and the role of redox signalling in chronic kidney disease. Nat. Rev. Nephrol..

[4] Rajaram S., Jones J., Lee G.J. (2019). Plant-based dietary patterns, plant foods, and age-related cognitive decline. Adv. Nutr..

[5] Qu G., Chen J., Guo X. (2019). The beneficial and deleterious role of dietary polyphenols on chronic degenerative diseases by regulating gene expression. Biosci. Trends.

[6] Khemka S., Reddy A., Garcia R.I., Jacobs M., Reddy R.P., Roghani A.K., Pattoor V., Basu T., Sehar U., Reddy P.H. (2023). Role of diet and exercise in aging, Alzheimer’s disease, and other chronic diseases. Ageing Res. Rev..

